# Satellite glial cell P2Y_12 _receptor in the trigeminal ganglion is involved in lingual neuropathic pain mechanisms in rats

**DOI:** 10.1186/1744-8069-8-23

**Published:** 2012-03-30

**Authors:** Ayano Katagiri, Masamichi Shinoda, Kuniya Honda, Akira Toyofuku, Barry J Sessle, Koichi Iwata

**Affiliations:** 1 Department of Physiology, Nihon University School of Dentistry, 1-8-13 Kandasurugadai, Chiyoda-ku, Tokyo 101-8310, Japan; 2 Department of Psychosomatic Dentistry, Tokyo Medical and Dental University Graduate School, 1-5-45 Yushima, Bunkyo-ku, Tokyo 113-8549, Japan; 3 Division of Functional Morphology, Dental Research Center, Nihon University School of Dentistry, 1-8-13 Kandasurugadai, Chiyoda-ku, Tokyo 101-8310, Japan; 4 Department of Oral and Maxillofacial Surgery, Nihon University School of Dentistry, 1-8-13 Kandasurugadai, Chiyoda-ku, Tokyo 101-8310, Japan; 5 Department of Oral Physiology, Faculty of Dentistry, University of Toronto, 124 Edward Street, Toronto, Ontario M5G 1G6, Canada; 6 Division of Applied System Neuroscience Advanced Medical Research Center, Nihon University Graduate School of Medical Science, 30-1 Ohyaguchi-Kamimachi, Itabashi, Tokyo 173-8610, Japan

**Keywords:** Neuron-Glia interactions, Lingual nerve injury, Mechanical allodynia, Heat hyperalgesia, Purinergic receptor

## Abstract

**Background:**

It has been reported that the P2Y_12 _receptor (P2Y_12_R) is involved in satellite glial cells (SGCs) activation, indicating that P2Y_12_R expressed in SGCs may play functional roles in orofacial neuropathic pain mechanisms. However, the involvement of P2Y_12_R in orofacial neuropathic pain mechanisms is still unknown. We therefore studied the reflex to noxious mechanical or heat stimulation of the tongue, P2Y_12_R and glial fibrillary acidic protein (GFAP) immunohistochemistries in the trigeminal ganglion (TG) in a rat model of unilateral lingual nerve crush (LNC) to evaluate role of P2Y_12_R in SGC in lingual neuropathic pain.

**Results:**

The head-withdrawal reflex thresholds to mechanical and heat stimulation of the lateral tongue were significantly decreased in LNC-rats compared to sham-rats. These nocifensive effects were apparent on day 1 after LNC and lasted for 17 days. On days 3, 9, 15 and 21 after LNC, the mean relative number of TG neurons encircled with GFAP-immunoreactive (IR) cells significantly increased in the ophthalmic, maxillary and mandibular branch regions of TG. On day 3 after LNC, P2Y_12_R expression occurred in GFAP-IR cells but not neuronal nuclei (NeuN)-IR cells (i.e. neurons) in TG. After 3 days of successive administration of the P2Y_12_R antagonist MRS2395 into TG in LNC-rats, the mean relative number of TG neurons encircled with GFAP-IR cells was significantly decreased coincident with a significant reversal of the lowered head-withdrawal reflex thresholds to mechanical and heat stimulation of the tongue compared to vehicle-injected rats. Furthermore, after 3 days of successive administration of the P2YR agonist 2-MeSADP into the TG in naïve rats, the mean relative number of TG neurons encircled with GFAP-IR cells was significantly increased and head-withdrawal reflex thresholds to mechanical and heat stimulation of the tongue were significantly decreased in a dose-dependent manner compared to vehicle-injected rats.

**Conclusions:**

The present findings provide the first evidence that the activation of P2Y_12_R in SGCs of TG following lingual nerve injury is involved in the enhancement of TG neuron activity and nocifensive reflex behavior, resulting in neuropathic pain in the tongue.

## Background

Neuropathic pain occurs and persists in a heterogeneous group of etiologically different diseases involving a peripheral nerve lesion or dysfunction of the peripheral or central nervous system. Neuropathic pain is relatively common and frequently resistant to clinical treatment [1].

Injury to trigeminal nerve branches is known to cause neuropathic pain in the orofacial region [2,3]. The lingual nerve, a branch of the trigeminal nerve, innervates the mucous membrane of the floor of the mouth, the lingual side of gingiva and the anterior two-thirds of the tongue. The lingual nerve is susceptible to iatrogenic damage during various surgical procedures such as removal of impacted lower third molar teeth or placement of dental implants, and damage to the nerve can results in temporary or permanent sensory abnormalities including mechanical allodynia and heat hyperalgesia [4-7].

In the trigeminal ganglion (TG), satellite glial cells (SGCs) encircle the somata of neurons [8], and have important functions in nourishing and supporting primary neurons [9-11]. Moreover, it has been reported that SGCs are activated [12] in response to peripheral nerve injury [13], and are involved in modulation of neuronal excitability [10,14]. However, the mechanisms underlying possible interaction between SGCs and TG neurons following peripheral nerve injury are still unknown.

Many substances are released from injured and uninjured neurons, such as nitric oxide, tumor necrosis factor-α and adenosine triphosphate (ATP) after peripheral nerve injury [15-18]. ATP is one of the most abundant neurotransmitters in the somatosensory system [19,20], and is involved in pain-related processing through the activation of metabotropic (P2Y family) or ionotropic (P2X family) purinergic receptors. SGCs in TG are known to express several purinergic receptors such as P2Y_1_R, P2Y_2_R, P2Y_4_R and P2Y_12_R subtypes [21-25].

These previous reports suggest that ATP released from TG neurons after lingual nerve injury may bind P2Y_12_R in SGCs, resulting in modulation of TG neuronal activity. Therefore, we investigated mechanical- and heat-evoked nocifensive reflex behaviors, SGC activation in TG, P2Y_12_R expression in SCGs, effect of P2YR agonist or P2Y_12_R antagonist on the nocifensive reflex behavior and SCG activation in the lingual nerve crushed (LNC)-rats, to determine if P2Y_12_R signaling in SGCs of TG is involved in lingual neuropathic pain mechanisms.

## Results

### Nocifensive reflex to mechanical or heat stimulation of the tongue

**Figure 1:**
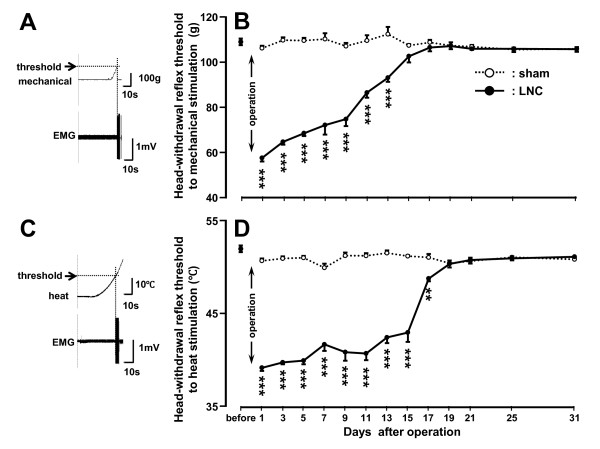
**Mechanical allodynia and heat hyperalgesia after LNC**. EMG activity recorded from the splenius capitis muscle during mechanical (A) or heat (C) stimulation of the tongue. Time-course changes are shown in mean head-withdrawal reflex threshold to mechanical stimulation of the ipsilateral tongue to in LNC- or sham-rats (B) and head-withdrawal reflex threshold to heat stimulation of the ipsilateral tongue to in LNC- or sham-rats (D). Head-withdrawal reflex threshold is indicated by the arrows in A and C. **: *p <*0.01, ***: *p *< 0.001 (vs. sham-rats; n = 7 in each group, two-way ANOVA with repeated measures, followed by Bonferroni's multiple-comparison tests).

The head-withdrawal reflex threshold to mechanical stimulation of the ipsilateral tongue significantly decreased from 1 to 13 days after LNC compared with that of sham-rats (*p *< 0.001, n = 7 in each group) (Figure 1B). The head-withdrawal reflex threshold to heat stimulation also significantly decreased from 1 to 17 days after LNC compared with that of sham-rats (*p *< 0.01, n = 7 in each group) (Figure 1D). There were no significant changes in the head-withdrawal reflex thresholds to mechanical and heat stimulation in sham-rats compared to naïve rats before operation during the experimental period. No significant changes in the head-withdrawal reflex thresholds to mechanical and heat stimulation of the contralateral tongue were observed in LNC-rats compared to naïve rats (data not shown).

### GFAP expression in TG

**Figure 2:**
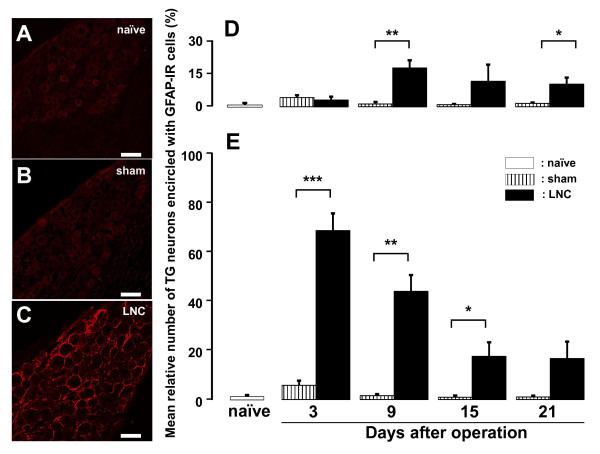
**GFAP expression in TG after LNC**. Photomicrographs of TG neurons encircled with GFAP-IR cells in the V3 branch region in naïve rats and those on day 3 after sham operation or LNC (A: naïve, B: sham, C: LNC). Time-course changes are shown in the mean relative number of TG neurons encircled with GFAP-IR cells on days 3, 9, 15 and 21 after sham operation or LNC. D: V1/V2 branch regions in TG, E: V3 branch region in TG. *: *p <*0.05, **: *p <*0.01, ***: *p *< 0.001 (n = 5 in each group, Student's *t*-test). Scale bars = 50 μm.

Glial fibrillary acidic protein (GFAP)-immunoreactive (IR) cells were not observed in TG in naïve and sham-rats (Figure 2A and 2B); however, numerous TG neurons encircled with GFAP-IR cells were seen in LNC-rats (Figure 2C). The mean relative number of TG neurons encircled with GFAP-IR cells (*p *< 0.05, cell numbers: naïve = 1757; sham = 5140; LNC = 4390; n = 5 rats in each group) was significantly increased on days 9 and 21 after LNC in the ophthalmic (V1) and maxillary (V2) branch regions of TG (Figure 2D). The mean relative number of TG neurons encircled with GFAP-IR cells (cell numbers: naïve = 1625; sham = 5191; LNC = 4516; n = 5 rats in each group) was also significantly increased in mandibular (V3) branch region of TG on days 3, 9 and 15 after LNC (Figure 2E).

**Figure 3:**
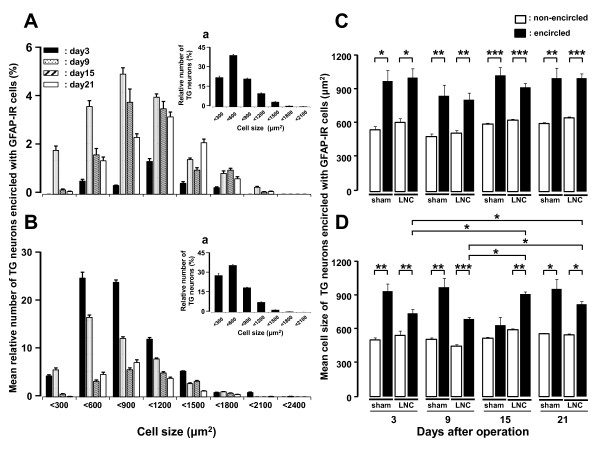
**Change in GFAP expression in TG after LNC**. Time-course changes are shown in the cell size of somata of TG neurons encircled with GFAP-IR cells on day 3, 9, 15 and 21 after LNC and sham operation in V1/V2 branch regions (A and C) and V3 branch region (B and D). Size-frequency histogram illustrating distribution of sotama of TG neurons encircled with GFAP-IR cells in V1/V2 branch regions (A) and V3 branch region (B) on days 3, 9, 15 and 21 after LNC. Size-frequency histograms illustrating distribution of somata of TG neurons in V1/V2 branch regions (Aa) and V3 branch region (Ba) in naïve rats, respectively. *: *p <*0.05, **: *p <*0.01, ***: *p *< 0.001 (n = 5 in each group, Student's *t*-test, one-way ANOVA followed by Newman-keuls tests).

To further characterize the subset of TG neurons encircled with GFAP-IR cells after LNC, we examined daily size-frequency histograms illustrating distribution of somata of TG neurons encircled with GFAP-IR cells in LNC-rats. Numerous somata of TG neurons encircled with GFAP-IR cells ranged from 600 to 1200 μm^2 ^in V1/V2 branch regions on days 3, 9, 15 and 21 after LNC (day 3: 57%, day 9: 53%, day 15: 65%, day 21: 55%) (Figure 3A). In V3 branch region, most of somata of TG neurons encircled with GFAP-IR cells had areas less than 600 μm^2 ^on days 3 and 9 (day 3: 40%, day 9: 48%), on the other hand those were ranging from 600 to 1200 μm^2 ^on days 15 and 21 (day 15: 58%, day 21: 63%) (Figure 3B). Furthermore, the mean cell size of TG neurons encircled with GFAP-IR cells was significantly larger than that of TG neurons non-encircled with GFAP-IR cells on days 3, 9, 15 and 21 after LNC and sham operation in the V1/V2 and V3 branch regions (*p *< 0.05, cell numbers: sham V1/V2 = 5140; LNC V1/V2 = 4390; sham V3 = 5191; LNC V3 = 4516; n = 5 rats in each group) (Figure 3C and 3D). The mean cell size of TG neurons encircled with GFAP-IR cells was significantly larger in V3 branch region on days 15 and 21 compared with that on days 3 and 9 after LNC as illustrated in Figure 3D (day 3 vs. day 15 or 21; day 9 vs. day 15 or 21, *p *< 0.05). We also observed that the mean cell size of TG neurons encircled with GFAP-IR cells was significantly larger than non-encircled neurons in sham-operated rats in V3 branch region on days 3, 9 and 21.

### P2Y_12_R expression and effect of administration of MRS2395 on GFAP expression in TG

**Figure 4:**
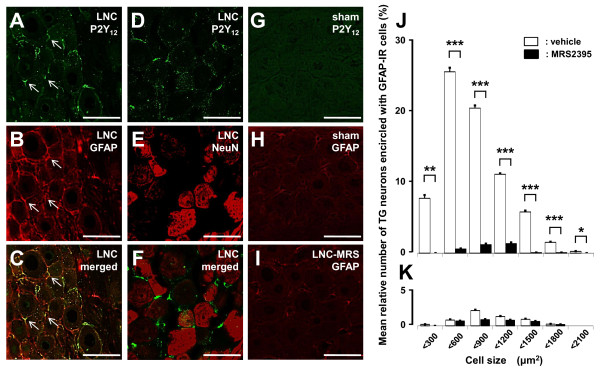
**Expression of P2Y_12_R and GFAP in TG and effect of P2Y_12_R antagonist on GFAP expression in LNC-rats**. Photomicrographs of P2Y_12_R-IR cells (A and D); GFAP-IR cells (B); P2Y_12_R-IR and GFAP-IR cells (C); NeuN-IR cells (E); P2Y_12_R-IR and NeuN-IR cells (F) in V3 branch region on day 3 after LNC. Photomicrographs of P2Y_12_R-IR cells (G); GFAP-IR cells (H) in V3 branch region on day 3 after sham operation. Photomicrographs of GFAP-IR cells following MRS2395 administration for 3 successive days into TG in LNC-rats (I). J and K: Size-frequency histograms illustrating distribution of somata of TG neurons encircled with GFAP-IR cells in V3 branch region on day 3 after operation and following daily successive MRS2395 (18.0 ng/day) or vehicle administration (from day 0 to day 2) into TG in LNC-rats (J) and sham-rats (K) on day 3 after LNC or sham operation. Arrows indicate GFAP-IR cells expressing P2Y_12_R-IR cells. *: *p <*0.05, **: *p <*0.01, ***: *p *< 0.001 (n = 5 in each group, Student's *t*-test). Scale bars = 50 μm.

We performed double immunohistochemical labeling for P2Y_12_R and GFAP on day 3 after LNC or sham operation. P2Y_12_R was expressed in GFAP-IR cells but not in neuronal nuclei (NeuN)-IR cells in TG in LNC-rats (Figure 4A-F). P2Y_12_R and GFAP were not expressed in TG in sham-rats (Figure 4G and 4H). A small number of GFAP-IR cells were observed in LNC-rats that had P2Y_12_R antagonist MRS2395 (Sigma-Aldrich, St. Louis, MI) administered for 3 successive days into TG as illustrated in Figure 4I, and the mean relative number of TG neurons encircled with GFAP-IR cells was significantly decreased in all cell size groups following 3 successive days of MRS2395 administration (18.0 ng in 0.5 μl/day) into TG in LNC-rats (*p *< 0.05, cell numbers: vehicle = 1530; MRS2395 = 1306; n = 5 rats in each group) (Figure 4J). There was no significant effect of MRS2395 administration on the mean relative number and cell size of TG neurons encircled with GFAP-IR cells in sham-rats (cell numbers: vehicle = 1936; MRS2395 = 1606; n = 5 rats in each group). There were no significant differences between specific size subgroups (Figure 4K).

### Effect of MRS2395 administration into TG on head-withdrawal reflex threshold

**Figure 5:**
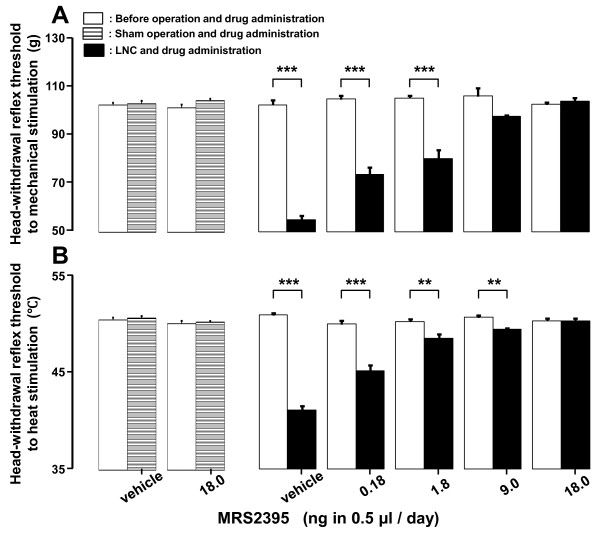
**Effect of P2Y_12_R antagonist on nocifensive reflex in LNC-rats**. Effect of daily successive administration (from day 0 to day 2) of MRS2395 or vehicle into TG on mean head-withdrawal reflex thresholds to mechanical (A) and heat (B) stimulation to the tongue in the LNC- or sham-rats on day 3 after operation. Head-withdrawal reflex threshold after LNC or sham operation with MRS2395 administration was compared with that before LNC or sham operation with MRS2395 administration. **: *p <*0.01, ***: *p *< 0.001 (n = 6 in each group, Student's *t*-test).

The decrement of head-withdrawal reflex thresholds to mechanical and heat stimulation of the tongue significantly reversed dose-dependently following successive MRS2395 administration (0.18, 1.8, 9.0 and 18.0 ng in 0.5 μl/day) for 3 days into TG after LNC (*p *< 0.01, n = 6 in each group) (Figure 5). MRS2395 administration for 3 days into TG did not change the head-withdrawal reflex thresholds to mechanical and heat stimulation of the tongue in sham-rats (n = 6 in each group) (Figure 5). No change in head-withdrawal reflex threshold was observed after cannula implantation alone (data not shown).

### Effect of administration of 2-MeSADP on GFAP expression in TG

**Figure 6:**
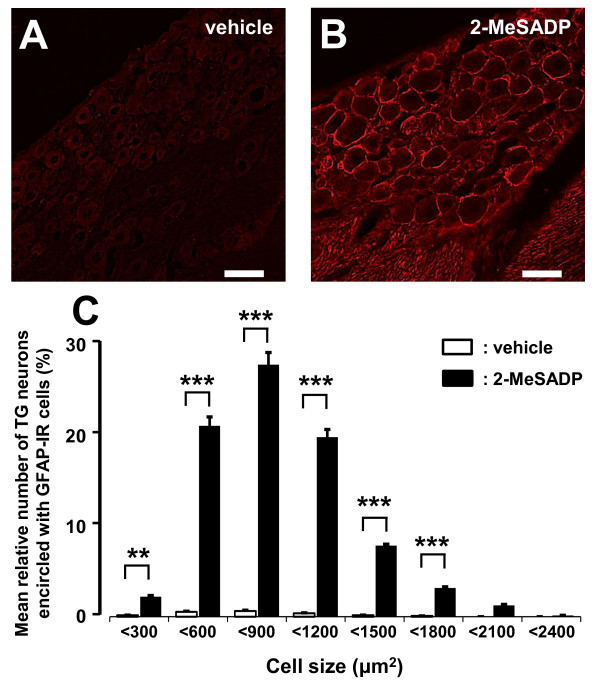
**Effect of P2YR agonist on GFAP expression in naïve rats**. Photomicrographs of GFAP-IR cells in V3 branch region following daily successive vehicle (A) or 2-MeSADP (B) administration (from day 0 to day 2) into TG on day 3 in naïve rats. C: The size-frequency histogram illustrating distribution of somata of TG neurons encircled with GFAP-IR cells in V3 region following successive daily 2-MeSADP (10.0 nmol/day) or vehicle administration (from day 0 to day 2) into TG on day 3 in naïve rats (C) **: *p <*0.01, ***: *p *< 0.001 (n = 5 in each group, Student's *t*-test). Scale bars = 50 μm.

In naïve rats, daily injection of the P2YR agonist 2-MeSADP (Sigma-Aldrich) administration (10.0 nmol in 0.5 μl/day from day 0 to day 2) into TG induced increased expression of GFAP-IR cells in TG (Figure 6A and 6B). The mean relative number of TG neurons encircled with GFAP-IR cells was significantly increased in naïve rats following successive 2-MeSADP administration for 3 days into TG compared to vehicle-administrated naïve rats (*p *< 0.01, cell numbers: vehicle = 1803, 2-MeSADP = 1006, n = 5 rats in each group) (Figure 6C).

### Effect of 2-MeSADP administration into TG on head-withdrawal reflex threshold

**Figure 7:**
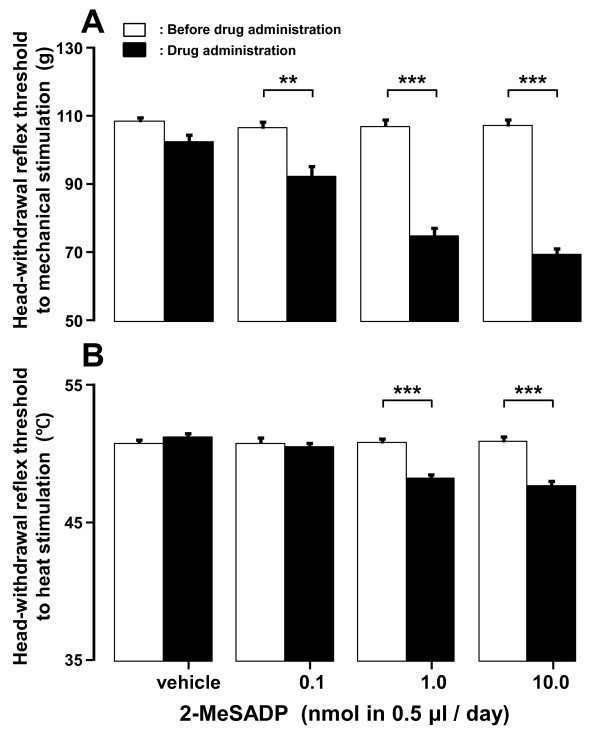
**Effect of P2YR agonist on nocifensive reflex in naïve rats**. Effect of vehicle or 2-MeSADP daily successive administration (from day 0 to day 2) into TG on mean mechanical (A) and heat (B) head-withdrawal reflex threshold on day 3 in naïve rats. Head-withdrawal reflex threshold after 2-MeSADP administration was compared with that before 2-MeSADP administration. **: *p <*0.01, ***: *p *< 0.001 (n = 6 in each group, Student's *t*-test).

Following successive 2-MeSADP administration (0.1, 1.0 and 10.0 nmol in 0.5 μl/day) for 3 days into TG in naïve rats, the head-withdrawal reflex thresholds to mechanical and heat stimulation of the tongue were significantly decreased in a dose-dependent manner (*p *< 0.01, n = 6 in each group) (Figure 7A and 7B).

### P2Y_1_R and P2Y_13_R expression in TG and effect of 2-MeSADP and MRS2395 administration into TG on head-withdrawal reflex threshold

We also performed immunohistochemical analysis for P2Y_1_R, P2Y_13_R, GFAP or NeuN in naïve, sham- or LNC-rats. P2Y_1_R was expressed in GFAP-IR and NeuN-IR cells in TG in naïve, sham- and LNC-rats (Additional file 1: Figure S1A-T). The number of TG neurons encircled with P2Y_1_R-IR cells and the number of P2Y_1_R-IR TG neurons were not significantly different among naïve, sham- and LNC-rats. P2Y_13_R was also expressed in NeuN-IR cells but not in GFAP-IR cells in TG in naïve, sham- or LNC-rats (Additional file 2: Figure S2A-S). The number of TG neurons encircled with P2Y_13_R-IR cells was not counted since we could not observe any TG neurons encircled with P2Y_13_R-IR cells. The number of P2Y_13_R-IR TG neurons was not significantly different among naïve, sham- and LNC-rats.

Following successive simultaneous administration of 2-MeSADP (10.0 nmol in 0.5 μl/day) and MRS2395 (18.0 ng in 0.5 μl/day) into TG for 3 days in naïve rats, the head-withdrawal reflex thresholds to mechanical and heat stimulation of the tongue were not changed (n = 6 in each group) (Additional file 3: Figure S3).

## Discussion

We studied the involvement of SGC-P2Y_12_R in tongue neuropathic pain following unilateral LNC. The nocifensive reflex thresholds were significantly decreased and the number of TG neurons encircled with GFAP-IR cells significantly increased in TG in LNC-rats. P2Y_12_R expression occurred in GFAP-IR cells in TG. After administration of the P2Y_12_R antagonist into TG in LNC-rats, the number of TG neurons encircled with GFAP-IR cells was significantly decreased in association with a significant reversal of the lowered nocifensive reflex thresholds to mechanical and heat stimulation of the tongue. Furthermore, after administration of the P2YR agonist into TG in naïve rats, the number of TG neurons encircled with GFAP-IR cells was significantly increased, and the nocifensive reflex threshold was significantly decreased. The present findings suggest that the activation of SGC-P2Y_12_R in TG plays a crucial role in tongue neuropathic pain following lingual nerve injury.

### Technical considerations

Behavioral testing is necessary to better define if the model used in the present study is appropriate for chronic lingual neuropathic pain [26]. Mechanical nocifensive behavior was tested for evidence of mechanical allodynia, and heat nocifensive behavior was assessed for heat hyperalgesia [27]. Since it is not possible to measure adequately nocifensive behavior associated with trigeminal nerve injury involving intraoral structures, we measured the head-withdrawal reflex in lightly anesthetized rats. The head-withdrawal reflex thresholds to mechanical and heat stimulation of the tongue were measured before the cannula implantation to allow for subsequent drug administration into TG to test if the cannula implantation affects the reflex threshold. No change in head-withdrawal reflex threshold was observed after cannula implantation alone, indicating that the cannula implantation procedure does not comprehensive the study of GFAP-IR cell expression and head-withdrawal reflex.

Furthermore, MRS2395 has been reported to exert specific antagonistic effects on P2Y_12_R in rat models with sciatic nerve ligation [28,29], thus we used MRS2395 as selective antagonist for P2Y_12_R [30] in TG. On the other hand, 2-MeSADP has been known as the agonist for P2Y_1_R and P2Y_13_R as well as P2Y_12_R [31].

### Lingual neuropathic pain model

The lingual nerve is susceptible to iatrogenic damage, that often results in neuropathic pain [5-7,32]. In our LNC model, lingual nerve was injured directly similar to lingual nerve injury in clinic. Therefore, the present LNC model could be defined as the posttraumatic neuropathic pain model after lingual nerve injury.

We observed a significant decrease in the head-withdrawal reflex thresholds to mechanical and heat stimulation of the tongue ipsilateral to the LNC compared with sham-rats. No significant changes in the head-withdrawal reflex thresholds to mechanical and heat stimulation of the tongue contralateral to the LNC were observed compared with the baseline before LNC. These observations suggest that the LNC induces mechanical allodynia and heat hyperalgesia in the tongue [14].

### TG neurons-SGCs interactions

Previous studies have demonstrated that a barrage of action potentials is generated in primary afferent fibers after peripheral nerve injury and that hyperexcitability of primary afferent neurons may play a crucial role in the initiation of injury-induced neuropathic pain [33,34]. A variety of molecules are released from the somata of dorsal root ganglion (DRG) [15] or TG neurons, and are thought to influence the activity of adjacent neurons via SGC-neuron or neuron-neuron interactions [24]. The neuronal somata encircled with SGCs are enclosed with a connective tissue sheath and forms a functional unit [10]. The distance between SGCs and neuronal surfaces is very small (about 20 nm), suggesting a close functional relationship [14,35], and indicating that activation of SGCs is likely the result of a secondary change driven by neuronal activity [36]. The significant increase in the number of TG neurons encircled with hyperactive SGCs was observed following LNC. Together with previous results, our data suggest that functional interactions between TG neurons and SGCs are involved in the enhancement of TG neuron excitability in LNC-rats.

ATP is one of the major transmitters mediating the neuron-SGCs communication [15], and is released from several sources after peripheral nerve injury, such as central terminals of the primary afferents, postsynaptic dorsal horn neurons, astrocytes or SGCs [20,24,37-40]. Furthermore, ATP causes an increase in intercellular Ca^2+ ^concentration in SGCs [21]. It had been reported that TG neurons and SGCs communicate through Ca^2+ ^signals and this form of intercellular signaling involves the activation of purinergic receptors. Additional mechanisms involve bidirectional neuron-SGC, signaling between SGCs and a switch from P2YR to P2XR in cultured TG cells in the face-inflamed mouse [40,41]. P2Y_12_R in SGCs of TG [22] produce an increase in Ca^2+ ^influx and K^+ ^outflow and thereby SGC activation [42].

We observed significant increase in the number of TG neurons encircled with GFAP-IR cells in the V1/V2 branch regions as well as V3 branch region following LNC indicating widespread effects on TG neurons. Furthermore, the significant decrease in the head-withdrawal reflex threshold to mechanical and heat stimulation of the tongue was observed after P2Y_1_, P2Y_12_R and P2Y_13_R agonist injection into TG in naïve rats, and the lowering the head-withdrawal reflex threshold was attenuated by P2Y_12_R antagonist in LNC rats. These observations strongly suggest that ATP has a crucial role for the activation of SGCs and those interactions with TG neurons following LNC.

### P2Y_12_R activation in SGCs

In other neuropathic pain models, administration of the P2Y_12_R antagonist MRS2395 or AR-C69931MX significantly alleviated mechanical allodynia and heat hyperalgesia [28,29,43]. In the present study, P2Y_12_R co-localized with most GFAP-IR cells, but not with neurons in TG. We also studied the effect of direct injection of MRS2395 or P2YR agonist 2-MeSADP into TG on the GFAP expression and head-withdrawal reflex thresholds to mechanical and heat stimulation of the tongue. Significant reductions of GFAP expression and significant reversal of head-withdrawal reflex threshold to mechanical stimulation were observed following MRS2395 administration for 3 days into TG in LNC-rats. Moreover, the number of TG neurons encircled with GFAP-IR cells was significantly increased, and head-withdrawal reflex thresholds to mechanical and heat stimulation of the tongue were significantly decreased following 2-MeSADP administration for 3 days into TG in naïve rats.

The expression of P2Y_1_R and P2Y_13_R was not modulated in LNC model, and the mechanical and heat head-withdrawal reflex threshold was not also affected by simultaneous injection of 2-MeSADP and MRS2395 into TG in naïve rats. However, P2Y_1_R-IR and P2Y_13_R-IR cells could be observed in TG as well as P2Y_12_R-IR cells. Thus, the involvement of P2Y_1_R and P2Y_13_R in mechanical allodynia and heat hyperalgesia could not be excluded in this model. P2Y_12_R was expressed in GFAP-IR cells in TG in LNC-rats, whereas P2Y_12_R and GFAP were not expressed in TG in naïve and sham-rats. Furthermore, a small number of GFAP-IR cells could be observed in LNC-rats and the decrement of the head-withdrawal reflex threshold to mechanical and heat stimulation was significantly reversed following successive MRS2395 administration into TG.

These findings suggest that ATP is released from the somata of TG neurons and bind P2Y_12_R in SGCs and result in activation of SGCs, consistent with the hypothesis that activated SGCs contribute to enhancement of nocifensive behavior in LNC-rats.

### Activation of SGCs encircling TG neurons

It has been reported that SGCs are localized around both injured and uninjured neurons in the DRG [14] and that SGCs are activated in response to peripheral nerve injury [13], resulting in modulation of neuronal excitability [10,14]. SGCs respond to the external stimulation with increases in the intracellular Ca^2+ ^level and transmit these calcium signals to adjacent non-stimulated SGCs as intracellular Ca^2+ ^waves, resulting in an increase in TG neuron excitability [44]. We observed an increase in the number of TG neurons encircled with GFAP-IR cells in the V1/V2 branch regions as well as V3 branch region following LNC, and a time difference in expression of GFAP was also observed between these three branch regions in TG. The peak was on day 3 after LNC in the V3 branch region, whereas it was on day 9 after LNC in the V1/V2 branch regions. After the peak, the increase of mean relative number of TG neurons encircled with GFAP-IR cells was gradually reduced. It is likely that activated SGCs in the V3 branch region have strong connections with those distributed in the V1/V2 branch regions in TG, and are involved in the activation of SGCs in the V1/V2 branch regions via the gap junctions. It has also been reported that variety of molecules such as ATP, SP, CGRP or NO are released from TG neurons affecting modulation of excitability of adjacent SGCs following peripheral nerve injury [14]. Activated SGCs are thought to be involved in activation of adjacent non-stimulated SGCs via Ca^2+ ^wave mechanism, suggesting that after LNC orofacial regions innervated by the V1/V2 branches become allodynic and hyperalgesic as well as these innervated by the V3 branch.

TG neurons have been classified into large (> 1000 μm^2^), medium (500 - 1000 μm^2^) and small (< 500 μm^2^) sized cells, each giving rise to Aβ, Aδ or C afferent fibers [45,46]. We observed a change in the distribution pattern of cell sizes of TG neurons encircled with GFAP-IR cells after LNC. In the V1/V2 branch regions, day 3: 57%, day 9: 53%, day 15: 65%, day 21: 55% of TG neurons ranging from 600 to 1200 μm^2 ^were encircled with GFAP-IR cells on day 3 to day 21 after LNC. Day 3: 40%, day 9: 48% of TG neurons encircled with GFAP-IR cells were ranging less than 600 μm^2 ^on day 3 and day 9 in V3 branch region, whereas day 15: 58%, day 21: 63% of TG neurons encircled with GFAP-IR were ranging from 600 to 1200 μm^2 ^on day 15 and day 21. In addition, the size of TG neurons encircled with GFAP-IR cells was significantly larger compared with those of non-encircled neurons. Furthermore in V3 branch region, the size of TG neurons encircled with GFAP-IR cells was significantly larger on days 15 and 21 compared with that on days 3 and 9 after LNC. Previous studies have reported that DRG neurons encircled with GFAP-IR cells have a wide range of sizes [47]. Based on the previous data and present results, Aβ or Aδ afferent fibers might be involved in the mechanical allodynia and heat hyperalgesia following LNC rather than C afferent fibers.

## Conclusions

This is the first documentation that lingual neuropathic pain is induced by lingual nerve injury in rats. Mechanical allodynia and heat hyperalgesia are obvious in this model, and the P2Y_12_R in SGCs of TG has an important role in the development of the neuropathic pain. SGCs in TG were strongly activated after LNC, and head-withdrawal reflex thresholds to mechanical and heat stimulation of the tongue were significantly reduced in LNC-rats. The expression of activated SGCs and lowering of the head-withdrawal reflex threshold in LNC-rats was significantly suppressed by administration of P2Y_12_R antagonist MRS2395 into TG, whereas SGC activation was significantly enhanced and head-withdrawal reflex thresholds to mechanical and heat stimulation of the tongue were significantly decreased following administration of P2YR agonist 2-MeSADP into TG in naïve rats. These findings suggest that lingual nerve injury may cause ATP release in TG and binding to the P2Y_12_R in SGCs, resulting in the activation of SGCs and subsequence reduction of the head-withdrawal reflex thresholds to mechanical and heat stimulation following LNC.

## Methods

### Animals

Male Sprague-Dawley rats (n = 212, Japan SLC, Shizuoka, Japan) weighing 200-300 g were used in this study. The animals were maintained in a temperature-controlled room (23°C) with a 12/12 hours light-dark cycle. Food and water were freely available. This study was approved by the Animal Experimentation Committee at the Nihon University. All surgery and animal care were conducted in accordance with the National Institutes of Health Guide for the Care and Use of Laboratory Animals and the guidelines for Institutional Animal Care, and the guidelines of the International Association for the Study of Pain [48].

### Surgery

Under anesthesia with an intraperitoneal (i.p.) injection of sodium pentobarbital (50 mg/kg; Schering Plough, Whitehouse Station, NJ), the rats were placed on a warm mat (37°C). An incision was made in the floor of the oral cavity beneath the tongue at the left side of lingual frenulum. The lingual nerve was exposed through the floor of the oral cavity and then crushed with an arterial clamp (30 g; Natsume, Tokyo, Japan) for 30 seconds, and then the incision was sutured after LNC. Sham-rats were operated with same procedure without performing the nerve crush.

### Head-withdrawal reflex threshold measurement

The thresholds for the head-withdrawal reflex to mechanical and heat stimulation of the lateral edge of tongue (3 mm posterior from tip of tongue) were measured, before and on days 1, 3, 5, 7, 9, 11, 13, 15, 17, 19, 21, 25 and 31 after the LNC or sham operation, under light anesthesia with 2% isoflurane (Mylan, Canonsburg, PA) in oxygen. Bipolar enamel-coated stainless steels wire electrodes (Narishige, Tokyo, Japan) were placed in the splenius capitis muscle for electromyogram (EMG) recording of the reflex response (interelectrode distance: 5-6 mm) [49].

Mechanical stimulation (0-130 g, 10 g/sec, cut off: 130 g) was applied to the lateral edge of the tongue ipsilateral to the LNC or sham operation by using forceps with flat tips (4 mm^2 ^square; Panlab s.l., Barcelona, Spain) in lightly anesthetized rats (n = 7 in each group). The tongue was pinched at dorsum linguae and hypoglottis (Additional file 4: Figure S4A). The stimulus velocity was manually controlled consecutively from 0 g to threshold values at a speed of 10 g/s. The threshold intensity for evoking EMG activity by mechanical stimulation of the tongue was defined as the mechanical head-withdrawal reflex threshold.

Heat stimulation (35-60°C, 1°C/sec, cut off: 60°C) was also applied to the lateral edge of the tongue ipsilateral to the LNC or sham operation by using a contact heat probe (9 mm^2 ^square; Intercross, Tokyo, Japan) in lightly anesthetized rats (n = 7 in each group). The threshold temperature for evoking EMG activity by heat stimulation to the tongue was defined as the heat head-withdrawal reflex threshold. The mechanical or heat stimulation was applied 3 times with 5 minute intervals, and the mean value of the head-withdrawal reflex thresholds were determined.

The baseline of the head-withdrawal reflex thresholds to mechanical and heat stimulation were measured in naïve rats before the LNC or sham operation.

### GFAP, P2Y_12_R, P2Y_1_R, P2Y_13_R and NeuN immunohistochemistries

On days 3, 9, 15 and 21 after the LNC or sham operation, rats were transcardially perfused with saline followed by a fixative containing 4% paraformaldehyde in 0.1 M phosphate buffer (pH 7.4) under 2% isoflurane in oxygen and sodium pentobarbital anesthesia (50 mg/kg, i.p., n = 5 in each group). LNC- and sham-rats received 2,2-dimethyl-propionic acid 3 -(2-chloro-6-methylaminopurin-9-yl)-2-(2,2-dimethyl-propionyloxymethyl)-propylester (MRS2395; 18.0 ng in 0.5 μl/day; Sigma-Aldrich) into TG for 3 successive days, and the naïve rats received 2-(Methylthio) adenosine5'-diphosphate trisodium salt hydrate (2-MeSADP; 10.0 nmol in 0.5 μl/day; Sigma-Aldrich) into TG for 3 successive days, and rats were transcardially perfused using a same fixative as described above (n = 5 in each group).

TGs in the ipsilateral side to LNC or sham operation, and left TGs in naïve rats were dissected out after perfusion and immersed in the same fixative for 4 h at 4°C, and then kept in 0.01 M phosphate-buffered saline (PBS) containing 20% sucrose (w/v) for 12 h for cryoprotection. The specimens were then embedded in Tissue Tek (Sakura Finetek, Torrance, CA) and stored until cryosectioning at -20°C. Ten μm TG sections were cut in the horizontal plane along the long axis. Every 15th section was thaw-mounted on MAS-GP micro slide glass (Matunami, Osaka, Japan) and dried overnight at room temperature. Four sections were chosen from each TG in each rat. These sections were processed for GFAP, P2Y_12_R, P2Y_1_R, P2Y_13_R and NeuN immunohistochemistries.

Sections were incubated with mouse anti-GFAP monoclonal antibody (Millipore, Billerica, MA) after dilution at a concentration of 1:800 in 0.01 M PBS containing 4% normal goat serum (NGS) and 0.3% Triton X-100 (Sigma-Aldrich) overnight at 4°C. After rinsing with 0.01 M PBS, sections were incubated in Alexa Fluor 568 anti-mouse IgG (1:200 in 0.01 M PBS; Invitrogen, Paisley, U.K.) for 2 h at room temperature. After rinsing with 0.01 M PBS, sections were coverslipped in mounting medium (Thermo Fisher Scientific, Fremont, CA) and examined under a fluorescence microscope and analyzed using a BZ-9000 system (Keyence, Osaka, Japan). No specific labeling was observed in the absence of primary antibody. The number and cell size of somata of TG neurons encircled with GFAP-IR cells over 2/3 of perimeters of somata of TG neurons were defined as TG neurons encircled with GFAP-IR cells (SensivMeasure; Mitani, Fukui, Japan). The number of TG neurons encircled with GFAP-IR cells was counted in each rat (n = 5 in each group) and the relative number of them was calculated by the following formula: 100 × number of neurons encircled with GFAP-IR cells/total number of neurons.

P2Y_12_R, P2Y_1_R, P2Y_13_R, GFAP and NeuN immunohistochemical analyses were conducted in naïve, sham- and LNC-rats to locate P2Y_12_R, P2Y_1_R and P2Y_13_R in TG (n = 5 in each group). Sections were incubated with rabbit anti-P2Y_12_R polyclonal antibody (1:200; Anaspec, Fremont, CA), rabbit anti-P2Y_1_R polyclonal antibody (1:300; Alomone labs, Jerusalem, Israel), rabbit anti-P2Y_13_R polyclonal antibody (1:300; Chemicon, Temicula, CA), mouse anti-GFAP monoclonal antibody (1:800; Millipore) and/or mouse anti-NeuN monoclonal antibody (1:1000; Chemicon) in 0.01 M PBS containing 4% NGS and 0.3% Triton X-100 (Sigma-Aldrich) overnight at 4°C. After rinsing with 0.01 M PBS, sections were incubated in Alexa Fluor 488 anti-rabbit IgG (1:200 in 0.01 M PBS; Invitrogen) and Alexa Fluor 568 anti-mouse IgG (1:200 in 0.01 M PBS; Invitrogen) for 2 h at room temperature. After rinsing with 0.01 M PBS, sections were coverslipped in mounting medium (Thermo Fisher Scientific) and examined under a fluorescence microscope.

The numbers of TG neurons encircled with P2Y_1_R-IR or P2Y_13_R-IR cells and P2Y_1_R-IR or P2Y_13_R-IR TG neurons were counted in naïve, sham- and LNC-rats. The relative numbers of them were calculated by the following formula: 100 × number of neurons encircled with P2Y_1_R-IR cells/total number of neurons; 100 × number of P2Y_1_R-IR or P2Y_13_R-IR TG neurons/total number of neurons.

### MRS2395 and 2-MeSADP administration into TG

Rats were anesthetized with 2% isoflurane in oxygen and sodium pentobarbital (50 mg/kg, i.p.) and placed in a stereotaxic apparatus. The skull was exposed and a small hole (diameter; 1 mm) was drilled above the location of the bifurcation between V1/V2 branch regions and V3 branch region of TG. The guide cannula was extended into the hole 9 mm below the skull surface into TG ipsilateral to the LNC or sham operation (2.8 mm anterior from lambda and 2.7 mm lateral to the midline) and was fixed to the skull with three stainless-steel screws and dental resin. The tip of the trocar was located just below the surface of V1/V2 branch region near the border between V3 and V1/V2 branches to inject drugs into TG. To define the position of the tip of the cannula, multiunit activities by mechanical stimulation of the V1/V2 face area were recorded by using the trocar as electrode. After completion of the surgery, penicillin G potassium (20,000 units; Meiji Seika, Tokyo, Japan) was injected intramuscularly to prevent infection. The rats were allowed to recover for 7 days before experiments were performed [44,50].

Rats were lightly anesthetized with 2% isoflurane in oxygen, a 31-gauge injection needle (Heraeus Kulzer Japan, Osaka, Japan) was inserted into TG through the guide cannula (positioned as described above and illustrated in Additional file 4: Figure S4B) after the trocar was removed. In advance, we confirmed the diffusion of dye into V3 branch region following 0.5 μl dye injection through the guide cannula. The injection needle was connected to the 10 μl Hamilton syringe to deliver 0.5 μl or 1.0 μl of drugs over a 30 seconds period. LNC- or sham-rats were administered vehicle (0.5 μl; dimethyl sulfoxide (25%) and polyethylene glycol 300 (75%)) or MRS2395 (0.18, 1.8, 9.0 and 18.0 ng in 0.5 μl/day; Sigma-Aldrich) dissolved in vehicle (n = 6 in each group) [29], and naïve rats were administrated saline (0.5 μl), 2-MeSADP (0.1, 1.0 and 10.0 nmol in 0.5 μl/day; Sigma-Aldrich) dissolved in saline, vehicle (1.0 μl; dimethyl sulfoxide (25%) and polyethylene glycol 300 (75%)) or 2-MeSADP (10.0 nmol in 0.5 μl/day) mixed with MRS2395 (18.0 ng in 0.5 μl/day) once a day into TG for 3 successive days (day 0 through day 2) (n = 6 in each group). Head-withdrawal reflex thresholds were then measured under light anesthesia with 2% isoflurane in oxygen.

### Statistical analysis

Data were expressed as means ± SEM. Statistical analyses were performed by Student's *t*-test, one-way analysis of variance (ANOVA) followed by Newman-keuls tests, or two-way repeated-measures ANOVA followed by Bonferroni's multiple comparison tests where appropriate. A value of *p *< 0.05 was considered as significant.

## Abbreviations

P2Y_12_R: P2Y_12 _receptor; P2Y_1_R: P2Y_1 _receptor; P2Y_13_R: P2Y_13 _receptor; SGC: satellite glial cell; GFAP: glial fibrillary acidic protein; TG: trigeminal ganglion; LNC: lingual nerve crush; IR: immunoreactive; NeuN: neuronal nuclei; ATP: adenosine triphosphate; V1: ophthalmic; V2: maxillary; V3: mandibular; MRS2395: (2,2-dimethyl-propionic acid 3 - (2-chloro-6-methylaminopurin-9-yl) - 2 - (2,2-dimethyl-propionyloxymethyl) - propylester; 2-MeSADP: (2-(Methylthio) adenosine5'-diphosphate trisodium salt hydrate; DRG: dorsal root ganglion; PBS: phosphate-buffered saline; NGS: normal goat serum; ANOVA: analysis of variance.

## Competing interests

The authors declare that they have no competing interests.

## Authors' contributions

All authors read and approved the final manuscript. AK and KH carried out the experiments and data analysis. AT helped the experiments, data analysis. BJS provided data interpretation and helped to finalize the manuscript. MS and KI conceptualized the hypothesis, designed and supervised the experiments, directed the data analysis, and finalized the manuscript.

## Supplementary Material

Additional file 1 **Figure S1 Expression of P2Y_1_R, GFAP and NeuN in TG**. Photomicrographs of P2Y_1_R-IR cells (A and K); GFAP-IR cells (B); P2Y_1_R-IR and GFAP-IR cells (C); NeuN-IR cells (L); P2Y_1_R-IR and NeuN-IR cells (M) in V3 branch region in naïve rats. Photomicrographs of P2Y_1_R-IR cells (D and N); GFAP-IR cells (E); P2Y_1_R-IR and GFAP-IR cells (F); NeuN-IR cells (O); P2Y_1_R-IR and NeuN-IR cells (P) in V3 branch region in sham-rats. Photomicrographs of P2Y_1_R-IR cells (G and Q); GFAP-IR cells (H); P2Y_1_R-IR and GFAP-IR cells (I); NeuN-IR cells (R); P2Y_1_R-IR and NeuN-IR cells (S) in V3 branch region on day 3 after LNC. Arrows indicate GFAP-IR or NeuN-IR cells expressing P2Y_1_R-IR cells. Scale bars = 50 μm. J: The mean relative number of TG neurons encircled with P2Y_1_R-IR cells in naive, sham- and LNC-rats. (n = 5 in each group). T: The mean relative number of P2Y_1_R-IR TG neurons in naive, sham- and LNC-rats. (n = 5 in each group). Click here for file

Additional file 2 **Figure S2 Expression of P2Y_13_R and NeuN in TG**. Photomicrographs of P2Y_13_R-IR cells (A and J); GFAP-IR cells (B); P2Y_13_R-IR and GFAP-IR cells (C); NeuN-IR cells (K); P2Y_13_R-IR and NeuN-IR cells (L) in V3 branch region in naïve rats. Photomicrographs of P2Y_13_R-IR cells (D and M); GFAP-IR cells (E); P2Y_13_R-IR and GFAP-IR cells (F); NeuN-IR cells (N); P2Y_13_R-IR and NeuN-IR cells (O) in V3 branch region in sham-rats. Photomicrographs of P2Y_13_R-IR cells (G and P); GFAP-IR cells (H); P2Y_13_R-IR and GFAP-IR cells (I); NeuN-IR cells (Q); P2Y_13_R-IR and NeuN-IR cells (R) in V3 branch region on day 3 after LNC. Arrows indicate NeuN-IR cells expressing P2Y_13_R-IR cells. Scale bars = 50 μm. S: The mean relative number of P2Y_13_R-IR TG neurons in naïve, sham- and LNC-rats. (n = 5 in each group). Click here for file

Additional file 3 **Figure S3 Effect of P2YR agonist and antagonist on nocifensive reflex in naïve rats**. Effect of vehicle or 2-MeSADP administration with MRS2395 (from day 0 to day 2) into TG on mean mechanical (A) and heat (B) head-withdrawal reflex threshold on day 3 in naïve rats. Head-withdrawal reflex threshold after 2-MeSADP and MRS2395 administration was compared with that before administration. (n = 6 in each group). Click here for file

Additional file 4 **Figure S4 A: Photograph of mechanical stimulation of the tongue by forceps**. B: Photograph of TG. Arrow indicates the location of the needle was inserted. Click here for file
