# Supplementary figures and images for: Satellite glial cell P2Y12 receptor in the trigeminal ganglion is involved in lingual neuropathic pain mechanisms in rats
Source: Mol Pain. 2012 Mar 30;8:23. doi: 10.1186/1744-8069-8-23 (PMC3386019; doi:10.1186/1744-8069-8-23)

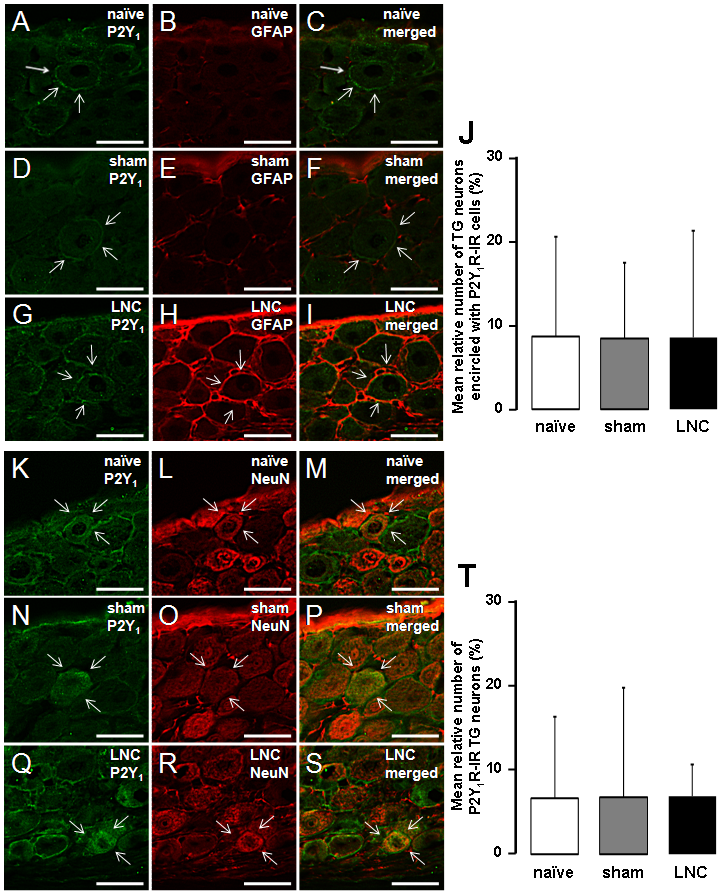

Supplement: Additional file 1 — Figure S1 Expression of P2Y1R, GFAP and NeuN in TG. Photomicrographs of P2Y1R-IR cells (A and K); GFAP-IR cells (B); P2Y1R-IR and GFAP-IR cells (C); NeuN-IR cells (L); P2Y1R-IR and NeuN-IR cells (M) in V3 branch region in naïve rats. Photomicrographs of P2Y1R-IR cells (D and N); GFAP-IR cells (E); P2Y1R-IR and GFAP-IR cells (F); NeuN-IR cells (O); P2Y1R-IR and NeuN-IR cells (P) in V3 branch region in sham-rats. Photomicrographs of P2Y1R-IR cells (G and Q); GFAP-IR cells (H); P2Y1R-IR and GFAP-IR cells (I); NeuN-IR cells (R); P2Y1R-IR and NeuN-IR cells (S) in V3 branch region on day 3 after LNC. Arrows indicate GFAP-IR or NeuN-IR cells expressing P2Y1R-IR cells. Scale bars = 50 μm. J: The mean relative number of TG neurons encircled with P2Y1R-IR cells in naive, sham- and LNC-rats. (n = 5 in each group). T: The mean relative number of P2Y1R-IR TG neurons in naive, sham- and LNC-rats. (n = 5 in each group). [file 1744-8069-8-23-S1.TIFF]

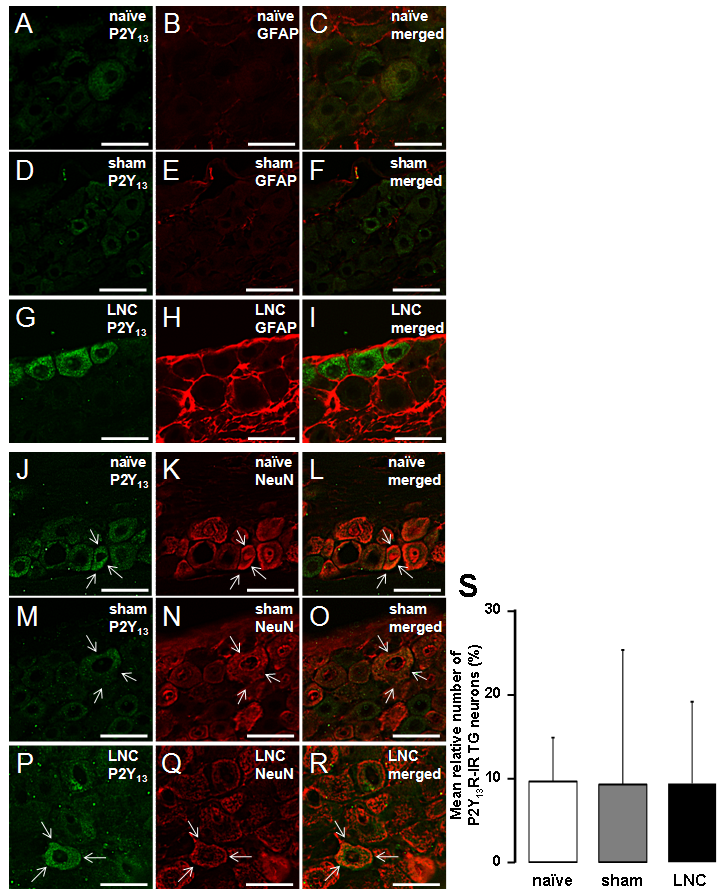

Supplement: Additional file 2 — Figure S2 Expression of P2Y13R and NeuN in TG. Photomicrographs of P2Y13R-IR cells (A and J); GFAP-IR cells (B); P2Y13R-IR and GFAP-IR cells (C); NeuN-IR cells (K); P2Y13R-IR and NeuN-IR cells (L) in V3 branch region in naïve rats. Photomicrographs of P2Y13R-IR cells (D and M); GFAP-IR cells (E); P2Y13R-IR and GFAP-IR cells (F); NeuN-IR cells (N); P2Y13R-IR and NeuN-IR cells (O) in V3 branch region in sham-rats. Photomicrographs of P2Y13R-IR cells (G and P); GFAP-IR cells (H); P2Y13R-IR and GFAP-IR cells (I); NeuN-IR cells (Q); P2Y13R-IR and NeuN-IR cells (R) in V3 branch region on day 3 after LNC. Arrows indicate NeuN-IR cells expressing P2Y13R-IR cells. Scale bars = 50 μm. S: The mean relative number of P2Y13R-IR TG neurons in naïve, sham- and LNC-rats. (n = 5 in each group). [file 1744-8069-8-23-S2.TIFF]

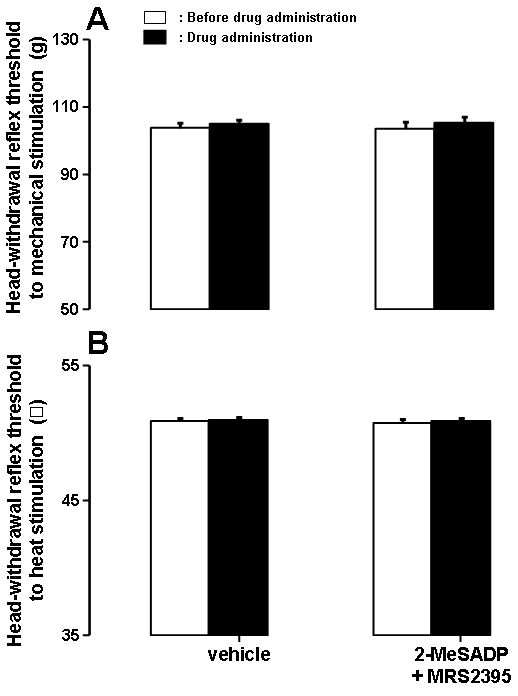

Supplement: Additional file 3 — Figure S3 Effect of P2YR agonist and antagonist on nocifensive reflex in naïve rats. Effect of vehicle or 2-MeSADP administration with MRS2395 (from day 0 to day 2) into TG on mean mechanical (A) and heat (B) head-withdrawal reflex threshold on day 3 in naïve rats. Head-withdrawal reflex threshold after 2-MeSADP and MRS2395 administration was compared with that before administration. (n = 6 in each group). [file 1744-8069-8-23-S3.TIFF]

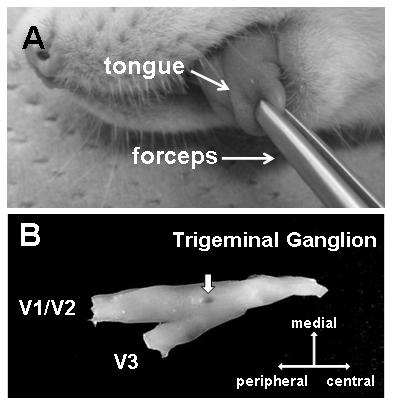

Supplement: Additional file 4 — Figure S4 A: Photograph of mechanical stimulation of the tongue by forceps. B: Photograph of TG. Arrow indicates the location of the needle was inserted. [file 1744-8069-8-23-S4.TIFF]
